# Home blood pressure-lowering effect of a non-steroidal mineralocorticoid receptor blocker, esaxerenone, versus trichlormethiazide for uncontrolled hypertension: the EXCITE-HT randomized controlled study

**DOI:** 10.1038/s41440-024-01762-z

**Published:** 2024-07-23

**Authors:** Kazuomi Kario, Hiroyuki Ohbayashi, Masami Hashimoto, Naoki Itabashi, Mitsutoshi Kato, Kazuaki Uchiyama, Kunio Hirano, Noriko Nakamura, Takahide Miyamoto, Hirotaka Nagashima, Shizuo Kajiyama, Hidenori Ishida, Enyu Imai, Yusuke Ebe, Mitsuru Ohishi, Tomohiro Katsuya, Takashi Taguchi, Ayumi Tanabe, Tatsuo Shimosawa, on behalf of the EXCITE-HT investigators

**Affiliations:** 1 Division of Cardiovascular Medicine, Department of Medicine, Jichi Medical University School of Medicine, Shimotsuke, Tochigi, Japan; 2 Tohno Chuo Clinic, Mizunami, Gifu, Japan; 3 Hashimoto Kidney Clinic, Fukuyama, Hiroshima, Japan; 4 Itabashi Diabetes and Dermatology Medical Clinic, Koga, Ibaraki, Japan; 5 Kato Clinic of Internal Medicine, Katsushika-ku, Tokyo, Japan; 6 Uchiyama Clinic, Joetsu, Niigata, Japan; 7 Hirano Clinic, Morioka, Iwate Japan; 8 Primula Clinic, Kagoshima, Kagoshima, Japan; 9 Miyamoto Clinic of Internal Medicine, Matsumoto, Nagano, Japan; 10 Tokyo Center Clinic, Chuo-ku, Tokyo, Japan; 11 Kajiyama Clinic, Kyoto, Kyoto, Japan; 12 Akaicho Clinic, Chiba, Chiba, Japan; 13 Nakayamadera Imai Clinic, Takarazuka, Hyogo Japan; 14 Ebe Clinic, Nagaoka, Niigata, Japan; 15 Department of Cardiovascular Medicine and Hypertension, Graduate School of Medical and Dental Sciences, Kagoshima University, Kagoshima, Kagoshima, Japan; 16 Katsuya Clinic, Amagasaki, Hyogo Japan; 17 Primary Medical Science Department, Medical Affairs Division, Daiichi Sankyo Co., Ltd., Chuo-ku, Tokyo, Japan; 18 Data Intelligence Department, Daiichi Sankyo Co., Ltd., Shinagawa-ku, Tokyo, Japan; 19 Department of Clinical Laboratory, School of Medicine, International University of Health and Welfare, Narita, Chiba, Japan

**Keywords:** Blood pressure, Esaxerenone, Essential hypertension, Japan, Trichlormethiazide

## Abstract

The EXCITE-HT study aimed to evaluate the efficacy and safety of esaxerenone versus thiazide diuretics (trichlormethiazide) as second-line treatment for Japanese patients with uncontrolled essential hypertension. This was a 12-week, multicenter, randomized, open-label, parallel-group study. The non-inferiority of esaxerenone to trichlormethiazide was confirmed if the upper limit of the two-sided 95% confidence interval (CI) for the difference in systolic blood pressure (SBP)/diastolic blood pressure (DBP) change between groups was below 3.9/2.1 mmHg. A total of 295 and 290 patients were included in the esaxerenone and trichlormethiazide groups, respectively. The non-inferiority of esaxerenone to trichlormethiazide was demonstrated: least squares mean change differences in morning home SBP/DBP at end of treatment (EOT) were −2.2 (95% CI, −3.6, −0.8) mmHg for SBP/−0.6 (−1.4, 0.2) mmHg for DBP. Morning home, bedtime home, and office BP significantly decreased (all *p* < 0.001) from baseline to EOT in both groups. The urinary albumin-to-creatinine ratio and N-terminal pro-brain natriuretic peptide level decreased from baseline to Week 12 in both groups, with no notable intergroup difference. Serum potassium elevations occurred more frequently with esaxerenone, while serum potassium reductions occurred more with trichlormethiazide. Uric acid elevations were observed in both groups, but more frequently with trichlormethiazide than esaxerenone. No cases of gout occurred in this study. Reductions in estimated glomerular filtration rate were similarly observed in both groups. EXCITE-HT is the first randomized controlled study to demonstrate evidence that esaxerenone is non-inferior to trichlormethiazide as second-line treatment for Japanese patients with uncontrolled essential hypertension, with no new safety concerns.

**Figure:**
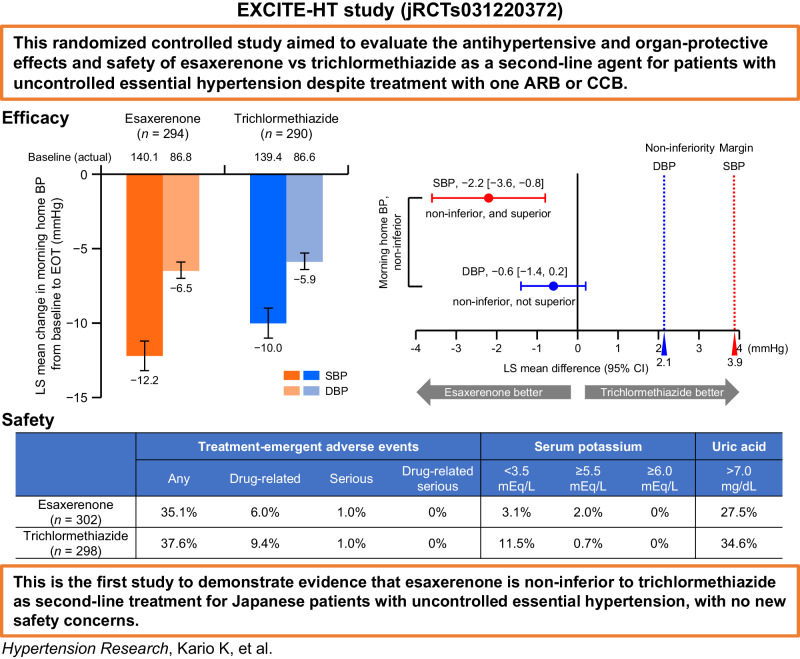
The EXCITE-HT study demonstrated the non-inferiority of esaxerenone to trichlormethiazide in its morning home blood pressure lowering effect and safety profile in Japanese patients with uncontrolled essential hypertension who were previously treated with an angiotensin II receptor blocker or calcium channel blocker.

The EXCITE-HT study demonstrated the non-inferiority of esaxerenone to trichlormethiazide in its morning home blood pressure lowering effect and safety profile in Japanese patients with uncontrolled essential hypertension who were previously treated with an angiotensin II receptor blocker or calcium channel blocker.

## Abstract (web summary)

The EXCITE-HT study demonstrated the non-inferiority of esaxerenone to trichlormethiazide in its morning home blood pressure lowering effect and safety profile in Japanese patients with uncontrolled essential hypertension who were previously treated with an angiotensin II receptor blocker or calcium channel blocker.

## Introduction

Hypertension is one of the main cardiovascular risk factors for cerebrovascular and cardiovascular events [1, 2], and adequate blood pressure (BP) control reduces these risks. Guidelines for hypertension management have been developed based on regional characteristics, and target BP levels for clinical practice have been clearly defined [3–6]. Additionally, many effective antihypertensive agents have been developed and are easily available in real-world clinical settings. However, adequate BP control remains an issue in some populations with hypertension [7]. In Korea and Taiwan, adequate BP control has been achieved in approximately 70% of the population, whereas in Japan, only 35% of the population has achieved adequate BP control; further improvement in hypertension management is still required [8]. Cardiovascular events are more closely associated with home BP, particularly morning home BP, rather than office BP [1, 2]. Currently, the 2019 Japanese Society of Hypertension (JSH) Guidelines recommend the use of home BP measurements for the diagnosis of hypertension and to evaluate the efficacy of antihypertensive medications [5]. Even among patients taking two or more antihypertensive agents, only 45% had adequate control of their morning home BP, which is considered trough BP [7]. The ‘hypertension paradox’ still exists, whereby the number of patients with uncontrolled BP does not decrease [9, 10]. One cause for this paradox is the limited BP-lowering effect of single agents, emphasizing the importance of antihypertensive agent combinations for patients with uncontrolled BP.

Esaxerenone is a next-generation non-steroidal mineralocorticoid receptor blocker (MRB) with higher selectivity and potency, longer half-life, and more favorable bioavailability compared with other MRBs [11, 12]. The MRB finerenone has shown renal event suppression, and in July 2021, it was approved by the US Food and Drug Administration for the treatment of chronic kidney disease associated with type 2 diabetes based on the results of the FIDELIO-DKD trial; however, in that study, finerenone did not show any significant BP-lowering effects [13]. Furthermore, unlike other MRBs, finerenone is not indicated as an antihypertensive drug in Japan, as no clinical trials have been conducted in hypertensive patients. Unlike spironolactone, esaxerenone has no reported adverse events of gynecomastia, but has a class effect of hyperkalemia [14]. In a phase 3 trial, esaxerenone 2.5 mg/day demonstrated non-inferiority to eplerenone 50 mg/day in BP-lowering effect, whereas esaxerenone 5 mg/day showed significantly greater reductions in BP compared with esaxerenone 2.5 mg/day [15]. Spironolactone is effective in patients with treatment-resistant hypertension when added as a fourth-line antihypertensive agent [16], whereas esaxerenone has shown a sustained 24-h antihypertensive effect when added as a second-line antihypertensive agent in a phase 3 trial [17, 18]. Furthermore, in post-marketing clinical studies, esaxerenone has shown favorable antihypertensive effects as a second-line antihypertensive agent in hypertensive patients with various characteristics [19–23]. Therefore, second-line treatment with esaxerenone may be a beneficial option for hypertensive patients, but there is currently no clinical evidence comparing esaxerenone with other antihypertensive agent classes.

Angiotensin II receptor blockers (ARBs) and calcium channel blockers (CCBs) are frequently prescribed as first-line treatment for hypertension. However, the clinical question remains as to which combination therapy is optimal when hypertension remains uncontrolled with these antihypertensive agents. Furthermore, most previous studies have focused on comparing antihypertensive agents used as first-line treatment; studies comparing antihypertensive agents used as second-line treatment are limited.

The EXCITE-HT study aimed to evaluate the antihypertensive and organ-protective effects and safety of esaxerenone versus trichlormethiazide as a second-line agent for patients with uncontrolled essential hypertension despite treatment with one ARB or CCB [24].

## Methods

### Study design

The EXCITE-HT study was a multicenter (54 sites) (Supplementary Table 1), randomized, open-label, parallel-group study conducted between December 2022 and September 2023. The study design details have been previously published [24]. This study was designed to determine the non-inferiority of esaxerenone versus trichlormethiazide in antihypertensive effects.

The study protocol was approved by the Certified Review Board of Hattori Clinic (CRB3180027) and was registered at the Japan Registry of Clinical Trials under the identifier jRCTs031220372. Written informed consent was obtained from patients before enrollment. The study was conducted in accordance with the principles of the Declaration of Helsinki and the Clinical Trials Act in Japan.

### Patients

Briefly, study participants who met all of the following criteria were included: age ≥20 years, prior treatment with either one ARB or one CCB at the same dose for ≥4 weeks prior to registration, and mean morning home systolic BP (SBP) ≥ 125 mmHg and/or diastolic BP (DBP) ≥ 75 mmHg. Patients with age ≥75 years, cerebrovascular disease, or proteinuria-negative chronic kidney disease were eligible if they had a mean morning home SBP of ≥135 mmHg and/or a DBP of ≥85 mmHg [24].

### Study interventions

Information on dose determination, criteria for concomitant drug use, and key discontinuation criteria have been previously reported [24]. Briefly, following a 4-week run-in period, esaxerenone (starting dose, 2.5 mg/day [patients with estimated glomerular filtration rate {eGFR} 30–59 mL/min/1.73 m^2^ or those with diabetes mellitus and albuminuria or proteinuria at baseline were administered 1.25 mg/day]; maximum dose, 5 mg/day) was administered for 12 weeks per the Japanese package insert [25]. Trichlormethiazide (recommended starting dose, ≤1 mg/day) was administered at the physician’s discretion per the Japanese package insert and the 2019 JSH guidelines [5, 26]. In both groups, the dose was increased at Weeks 4 and 8 according to the physician’s judgement based on the patient’s condition. Basal antihypertensive agents (ARBs or CCBs) were administered at a constant dose until the end of treatment (EOT), and the concomitant use of antihypertensive agents other than the assigned medication was prohibited.

### Study measurements

Home BP was measured twice daily in the morning and at bedtime using an upper arm cuff sphygmomanometer (HCR-7501T, OMRON Healthcare Co., Ltd., Japan). The averages of the two measurements at each timepoint within the last 5 days before the patient’s visit were recorded [24]. Office BP was measured twice and was averaged at each of the following timepoints: at baseline; Weeks 2, 4, 8, and 12; and at discontinuation [5, 24].

### Study endpoints

The primary endpoint was the change from baseline in morning home SBP/DBP to EOT. Secondary endpoints included change from baseline in bedtime home and office SBP/DBP to EOT, achievement rate of target BP levels, change from baseline in urinary albumin-to-creatinine ratio (UACR), and serum N-terminal pro-brain natriuretic peptide (NT-proBNP) levels at Week 12. The achievement rate of target BP levels was calculated by defining two criteria and determining the number of patients who met both the SBP and DBP targets [5].

Key safety endpoints included treatment-emergent adverse events (TEAEs); change from baseline in eGFR, uric acid (UA), blood electrolytes, and clinical laboratory test values; the proportion of patients with a serum potassium (K) level <3.5 mEq/L, ≥5.5 mEq/L, and ≥6.0 mEq/L; and the proportion of patients with a UA level >7.0 mg/dL.

### Sample size and statistical analyses

To verify the non-inferiority of esaxerenone to trichlormethiazide for the primary endpoint with sufficient statistical power (≥80% power, 2.5% one-sided type 1 error), the target sample size per treatment group was 270 [24]. The BP-lowering effect of esaxerenone was evaluated as non-inferior to that of trichlormethiazide if the upper limit of the two-sided 95% confidence interval (CI) for the difference in both SBP and DBP change between esaxerenone and trichlormethiazide was below 3.9 mmHg and 2.1 mmHg, respectively. Esaxerenone was considered superior to trichlormethiazide if the upper limit of the two-sided 95% CI was below 0. Multiplicity adjustments were made only for the primary endpoint. For the primary objective (non-inferiority validation of the primary efficacy endpoint), the main analysis was performed using the full analysis set (FAS), and an ancillary analysis was performed using the per protocol set (PPS) [24]. For the primary endpoint, the least squares (LS) mean, 95% CI, and the point estimate of the between-group difference were calculated using an analysis of covariance model. For BP, missing values at EOT were imputed by the last observation carried forward method using data from Week 4 onward. All statistical analyses have a two-sided significance level of 5% (unless otherwise stated) and were conducted using SAS version 9.4 or higher (SAS Institute Inc., Cary, NC, USA).

## Results

### Patients

Overall, 736 patients were assessed for eligibility during the 4-week run-in period, and 600 eligible patients were randomized; 295 and 290 patients (FAS) were included in the esaxerenone and trichlormethiazide groups, respectively (Supplementary Fig. 1). Baseline patient characteristics were similar between the two groups, with no statistical significance test (Table 1). Mean morning home, bedtime home, and office SBP/DBP were 140.1/86.8 and 139.4/86.6 mmHg, 134.7/81.5 and 134.4/81.3 mmHg, and 143.8/83.5 and 142.7/83.4 mmHg in the esaxerenone and trichlormethiazide groups, respectively. Baseline characteristics of the PPS are shown in Supplementary Table 2.

**Table 1:** Baseline patient characteristics (full analysis set)

Characteristics	Esaxerenone *n* = 295	Trichlormethiazide *n* = 290
Sex, male	149 (50.5)	160 (55.2)
Age, years	65.2 ± 11.5	64.7 ± 12.1
Weight, kg	65.66 ± 14.54	66.63 ± 13.90
Body mass index, kg/m^2^	25.38 ± 4.32	25.53 ± 4.20
Morning home SBP, mmHg	140.1 ± 15.0	139.4 ± 13.1
Morning home DBP, mmHg	86.8 ± 9.6	86.6 ± 9.4
Bedtime home SBP, mmHg	134.7 ± 15.8	134.4 ± 14.0
Bedtime home DBP, mmHg	81.5 ± 10.5	81.3 ± 10.8
Office SBP, mmHg	143.8 ± 16.5	142.7 ± 15.1
Office DBP, mmHg	83.5 ± 11.6	83.4 ± 12.2
NT-proBNP, pg/mL	109.49 ± 308.62 51.00 (0.5, 4229.0)	83.84 ± 147.38 46.00 (0.5, 1763.0)
<55	125 (42.4)	140 (48.3)
55 to <125	63 (21.4)	72 (24.8)
≥125	49 (16.6)	41 (14.1)
UACR, mg/gCr	116.27 ± 482.31 15.00 (2.0, 6600.0)	101.35 ± 424.41 17.95 (1.7, 6430.0)
<30	193 (65.4)	187 (64.5)
30 to <300	83 (28.1)	83 (28.6)
≥300	19 (6.4)	20 (6.9)
Serum K, mEq/L	4.21 ± 0.35	4.21 ± 0.32
Uric acid, mg/dL	5.39 ± 1.26	5.40 ± 1.20
eGFRcreat, mL/min/1.73 m^2^	71.50 ± 15.74	72.13 ± 17.09
Duration of hypertension, years	5.33 ± 5.08	5.46 ± 5.03
Complication	285 (96.6)	276 (95.2)
T2DM	119 (40.3)	115 (39.7)
Dyslipidemia	188 (63.7)	171 (59.0)
Hyperuricemia	45 (15.3)	44 (15.2)
Heart failure	22 (7.5)	16 (5.5)
Dose of esaxerenone at baseline (initial dose), mg		
1.25	118 (40.0)	–
2.5	177 (60.0)	–
Dose of esaxerenone at EOT (last dose), mg		
1.25	65 (22.0)	–
2.5	174 (59.0)	–
5	56 (19.0)	–
Dose of trichlormethiazide at baseline (initial dose), mg		
0.25	–	4 (1.4)
0.5	–	17 (5.9)
1	–	262 (90.3)
2	–	7 (2.4)
Dose of trichlormethiazide at EOT (last dose), mg		
0.25	–	2 (0.7)
0.5	–	18 (6.2)
1	–	244 (84.1)
>1 to ≤2	–	24 (8.3)
≥3	–	2 (0.7)
Basal antihypertensive agent		
ARB	119 (40.3)	116 (40.0)
CCB	176 (59.7)	174 (60.0)

Data are *n* (%), mean ± standard deviation, or median (minimum, maximum)

*ARB* angiotensin receptor blocker, *CCB* calcium channel blocker, *DBP* diastolic blood pressure, *eGFRcreat* creatinine-based estimated glomerular filtration rate, *EOT* end of treatment, *K* potassium, *NT-proBNP* N-terminal pro-brain natriuretic peptide, *SBP* systolic blood pressure, *T2DM* type 2 diabetes mellitus, *UACR* urinary albumin-to-creatinine ratio

### BP-lowering effects

**Fig. 1:**
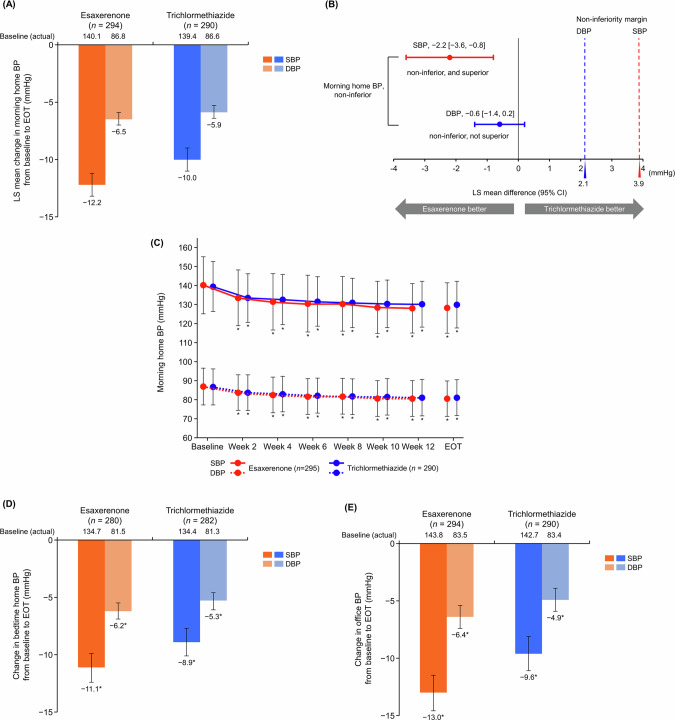
Changes from baseline to EOT in (**A**–**C**) morning home, (**D**) bedtime home, and (**E**) office BP (full analysis set). For panel **B**, the red dotted line (3.9 mmHg) and blue dotted line (2.1 mmHg) indicate the non-inferiority criteria. Data are LS mean (95% CI) for panels **A** and **B**. Data are arithmetic mean ± standard deviation for panel **C**. Data are arithmetic mean (95% CI) for panels **D** and **E**. *p < 0.001 versus baseline, paired *t-*test. BP blood pressure, CI confidence interval; DBP diastolic blood pressure, EOT end of treatment, LS least squares; SBP systolic blood pressure

Morning home SBP/DBP significantly decreased from baseline to EOT in both groups (Supplementary Table 3). The LS mean change in morning home SBP/DBP was a decrease of −12.2 (95% CI, −13.2, −11.2)/−6.5 (−7.0, −5.9) mmHg in the esaxerenone group and a decrease of −10.0 (−11.0, −9.0)/−5.9 (−6.4, −5.3) mmHg in the trichlormethiazide group (Fig. 1A). The primary endpoint, LS mean change differences in morning home SBP/DBP between the two groups at EOT were −2.2 (−3.6, −0.8)/−0.6 (−1.4, 0.2) mmHg (Fig. 1B). Upper limits of the two-sided 95% CIs for SBP and DBP were within the predefined noninferiority margin. The result showed the non-inferiority of esaxerenone to trichlormethiazide for both SBP and DBP, and superiority for SBP but not for DBP. Similar results were shown in the PPS (Supplementary Table 4). Therefore, esaxerenone demonstrated non-inferiority to trichlormethiazide in its antihypertensive effects based on morning home BP in both the FAS and PPS.

Morning home SBP/DBP decreased until Week 4 after starting esaxerenone treatment; this BP reduction was maintained until Week 12 and EOT (Fig. 1C). Significant reductions were also shown in bedtime home and office SBP/DBP in both treatment groups (all *p* < 0.001) (Fig. 1D, E and Supplementary Table 3). The PPS had similar results for morning and bedtime home and office BP (Supplementary Table 4).

The target BP level achievement rates at Week 12 in morning home BP were 60.8% and 55.8% in the esaxerenone and trichlormethiazide groups, respectively, for criteria 1, and 18.1% and 12.2% for criteria 2 (Table 2). Both groups showed similar tendencies in achievement rates for target BP level in morning home BP, bedtime home BP, and office BP. Similar trends were observed in the PPS (Supplementary Table 5).

**Table 2:** Achievement rate of target BP levels at Week 12 (full analysis set)

	Criteria 1 Home SBP/DBP of < 135/85 mmHg Office SBP/DBP of < 140/90 mmHg				Criteria 2 Home SBP/DBP of < 125/75 mmHg Office SBP/DBP of < 130/80 mmHg			
	Esaxerenone		Trichlormethiazide		Esaxerenone		Trichlormethiazide	
BP	*n*	Achievement rate	*n*	Achievement rate	*n*	Achievement rate	*n*	Achievement rate
Morning home BP	283	60.8 (54.8, 66.5)	285	55.8 (49.8, 61.6)	259	18.1 (13.6, 23.4)	263	12.2 (8.5, 16.7)
Bedtime home BP	280	77.1 (71.8, 81.9)	283	71.0 (65.4, 76.2)	256	37.5 (31.5, 43.7)	261	27.6 (22.3, 33.4)
Office BP	283	72.4 (66.8, 77.6)	284	64.8 (58.9, 70.3)	259	35.1 (29.3, 41.3)	262	28.2 (22.9, 34.1)

Data are % (95% CI) calculated using the Clopper–Pearson method

Criterion 1 was analyzed in all patients, and criterion 2 was analyzed in patients who were aged <75 years, or CKD (UACR ≥30 mg/gCr), or diabetes mellitus

*BP* blood pressure, *CI* confidence interval, *CKD* chronic kidney disease, *DBP* diastolic blood pressure, *SBP* systolic blood pressure, *UACR* urinary albumin-to-creatinine ratio

### UACR and NT-proBNP

**Fig. 2:**
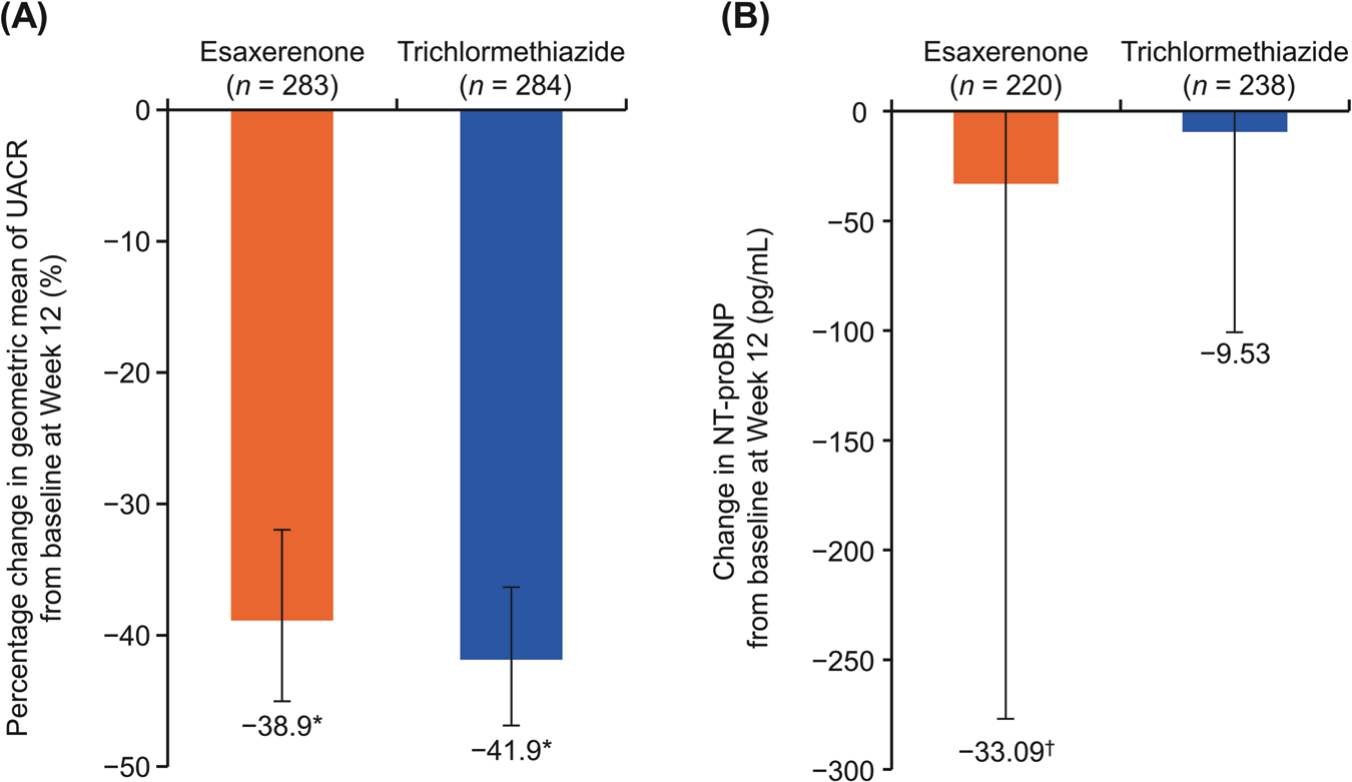
Percentage change in geometric mean of UACR (**A**) and change in NT-proBNP (**B**) during the study period (full analysis set). Data are mean (95% CI) for UACR and mean ± SD for NT-proBNP. *p < 0.001, ^†^*p* < 0.05 versus baseline, paired *t*-test. CI confidence interval, NT-proBNP N-terminal pro-brain natriuretic peptide, SD standard deviation, UACR urinary albumin-to-creatinine ratio

UACR decreased from baseline at Week 12 in the esaxerenone and trichlormethiazide groups (the percentage change in geometric mean, −38.9% and −41.9%, respectively; all *p* < 0.001 versus baseline) (Fig. 2A, Supplementary Table 6). Changes in NT-proBNP levels from baseline at Week 12 were −33.09 ± 243.79 and −9.53 ± 91.34 pg/mL in the esaxerenone and trichlormethiazide groups, respectively (Fig. 2B and Supplementary Table 6). Similar results were also observed in the PPS (Supplementary Table 7).

### Safety

In the esaxerenone and trichlormethiazide groups, TEAEs occurred in 35.1% and 37.6% of patients, respectively (Table 3). Drug-related TEAEs occurred in 18 (6.0%) and 28 (9.4%) patients, respectively, of whom one (0.3%) and five (1.7%) discontinued study treatment. Most TEAEs were mild or moderate in both treatment groups. Serious TEAEs were reported in three (1.0%) patients in each group; all were not related to the study drugs. Regarding TEAEs related to serum K, hypokalemia, blood K increased, and blood K decreased occurred in 0.3%, 2.0%, and 0% of patients in the esaxerenone group and in 1.3%, 0%, and 1.0% of patients in the trichlormethiazide group, respectively; no cases of hyperkalemia occurred in any of the groups.

**Table 3:** Summary of TEAEs (safety analysis set)

Type of TEAE	Esaxerenone *n* = 302	Trichlormethiazide *n* = 298
Any TEAEs	106 (35.1)	112 (37.6)
Drug-related TEAEs	18 (6.0)	28 (9.4)
Serious TEAEs	3 (1.0)	3 (1.0)
Drug-related serious TEAEs	0 (0.0)	0 (0.0)
Discontinued study treatment due to TEAEs	4 (1.3)	6 (2.0)
Discontinued study treatment due to drug-related TEAEs	1 (0.3)	5 (1.7)
Deaths	0 (0.0)	0 (0.0)
Frequent TEAEs occurring in ≥3 (1%) patients in either treatment group		
Nasopharyngitis	11 (3.6)	10 (3.4)
Hyperuricemia	4 (1.3)	11 (3.7)
Bronchitis	8 (2.6)	5 (1.7)
Dizziness	6 (2.0)	7 (2.3)
Blood potassium increased	6 (2.0)	0 (0.0)
COVID-19	5 (1.7)	5 (1.7)
Back pain	4 (1.3)	3 (1.0)
Headache	4 (1.3)	2 (0.7)
Diarrhea	4 (1.3)	2 (0.7)
Upper respiratory tract inflammation	4 (1.3)	0 (0.0)
Hypokalemia	1 (0.3)	4 (1.3)
Abdominal pain upper	1 (0.3)	4 (1.3)
Blood uric acid increased	1 (0.3)	4 (1.3)
Pharyngitis	3 (1.0)	3 (1.0)
Rhinitis allergic	3 (1.0)	2 (0.7)
Malaise	3 (1.0)	2 (0.7)
Gastroenteritis	3 (1.0)	1 (0.3)
Conjunctivitis allergic	3 (1.0)	1 (0.3)
Eczema	3 (1.0)	1 (0.3)
Urine albumin/creatinine ratio increased	3 (1.0)	1 (0.3)
Arthralgia	2 (0.7)	3 (1.0)
Myalgia	2 (0.7)	3 (1.0)
Glomerular filtration rate decreased	1 (0.3)	3 (1.0)
Type 2 diabetes mellitus	0 (0.0)	3 (1.0)
Dermatitis contact	0 (0.0)	3 (1.0)
Blood potassium decreased	0 (0.0)	3 (1.0)
Blood sodium decreased	0 (0.0)	3 (1.0)

Data are *n* (%). MedDRA/J version 25.1

*MedDRA* Medical Dictionary for Regulatory Activities, *TEAE* treatment-emergent adverse event

**Fig. 3:**
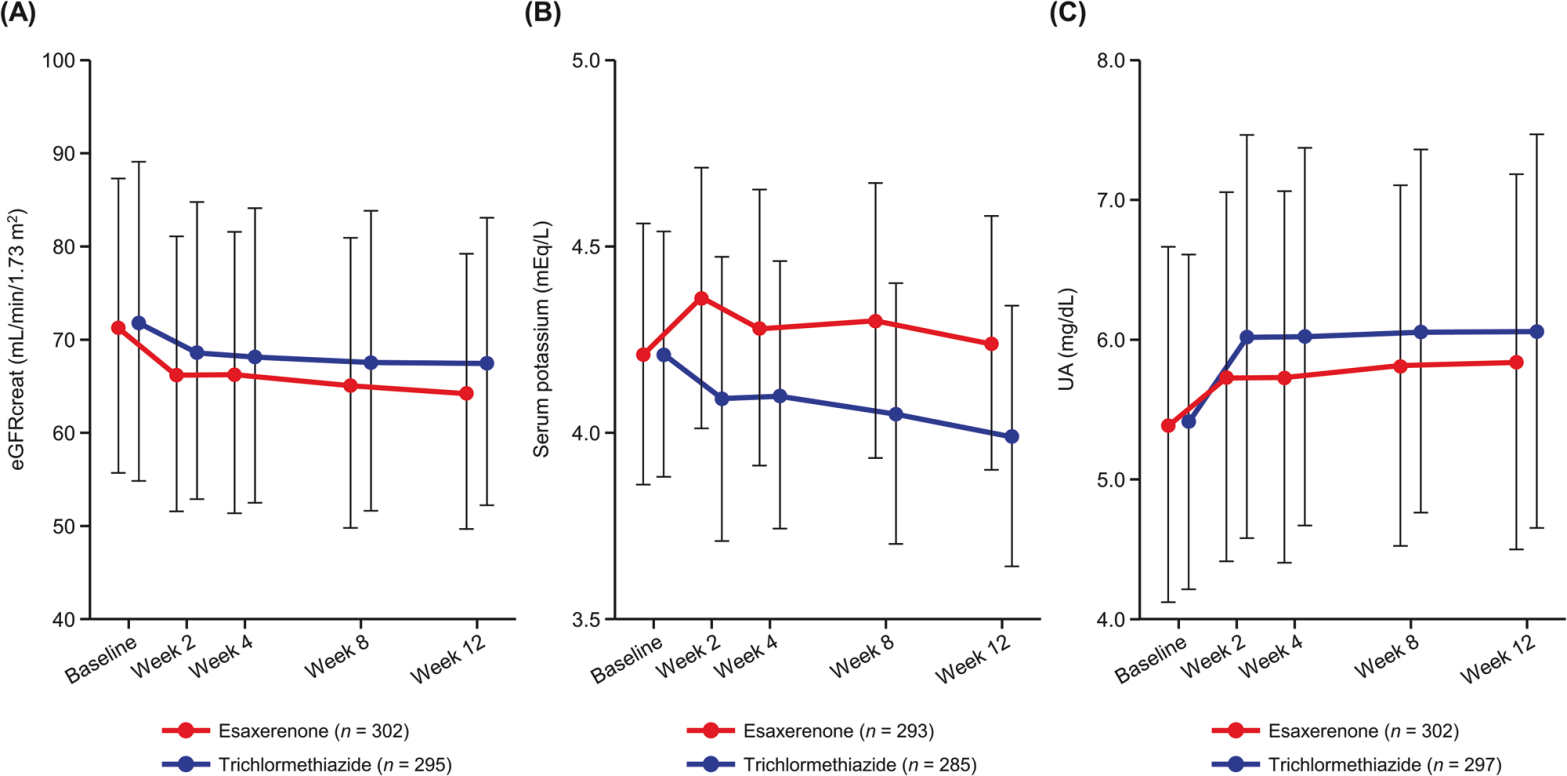
Time course changes in eGFRcreat (**A**), serum K levels (**B**), and UA (**C**) during the study period (safety analysis set). Data are mean ± standard deviation. eGFRcreat creatinine-based estimated glomerular filtration rate, K potassium, SD standard deviation, UA uric acid

eGFR decreased over the first 2 weeks and remained constant until Week 12 (Fig. 3A and Supplementary Table 8).

Serum K levels increased over the first 2 weeks after starting esaxerenone treatment, then gradually decreased up to Week 12 (Fig. 3B and Supplementary Table 8). Serum K levels gradually decreased in the trichlormethiazide group. The percentages of patients with serum K levels <3.5 mEq/L, ≥5.5 mEq/L, and ≥6.0 mEq/L were 3.1%, 2.0%, and 0% in the esaxerenone group and 11.5%, 0.7%, and 0% in trichlormethiazide group, respectively (Supplementary Table 9).

UA levels increased over the first 2 weeks, then remained constant until Week 12 in both groups (Fig. 3C and Supplementary Table 8). The percentages of patients with UA levels >7.0 mg/dL were 27.5% and 34.6% in the esaxerenone and trichlormethiazide groups, respectively. No cases of gout occurred in this study. Other biomarker data are shown in Supplementary Table 8.

## Discussion

The EXCITE-HT study is the first randomized controlled trial to compare the antihypertensive effect of esaxerenone as a second-line antihypertensive agent with another agent class in patients with uncontrolled essential hypertension. The non-inferiority of esaxerenone to trichlormethiazide in antihypertensive effects was demonstrated based on morning home BP. Target BP level achievement rates of esaxerenone were comparable to or higher than those of trichlormethiazide. The range of serum K elevation observed during esaxerenone treatment was similar to that of previous esaxerenone clinical studies [14, 19–23]. The validation of the non-inferior antihypertensive efficacy of esaxerenone compared with thiazide diuretics, one of the first-line agents according to guidelines [3–6], is important evidence for considering the early use of esaxerenone in hypertension treatment.

The change from baseline in morning home SBP/DBP was −12.2/−6.5 mmHg and −10.0/−5.9 mmHg in the esaxerenone and trichlormethiazide, with intergroup differences of −2.2 (95% CI, −3.6, −0.8)/−0.6 (−1.4, 0.2) mmHg. This reduction in morning home BP with esaxerenone was similar to that observed in the ENaK study [22]. It has been reported that a −2.5 mmHg reduction in morning home SBP contributes to a 3.5–9.5% reduction in cardiovascular disease risk [27]. Although no superiority was demonstrated for DBP, esaxerenone significantly lowered SBP by −2.2 mmHg versus trichlormethiazide, indicating a clinically meaningful reduction. In the NOCTURNE study, the addition of 1 mg trichlormethiazide to an ARB led to a change in morning home SBP/DBP of −9.4/−4.8 mmHg [28]. In the present study, trichlormethiazide was prescribed at a low dose according to the guidelines, but the antihypertensive effect on SBP was comparable to that in the NOCTURNE study [28], whereas a stronger antihypertensive effect on DBP by 1 mmHg was confirmed. The degree of BP reduction with antihypertensive agents is more pronounced in patients with untreated and/or severe hypertension. In the present study, baseline morning home SBP/DBP values were 140.1/86.8 mmHg for esaxerenone and 139.4/86.6 mmHg for trichlormethiazide, indicating that the majority of patients had mild hypertension. This patient background characteristic should be noted in comparisons with other clinical studies.

The achievement rate of target morning home BP levels (SBP/DBP < 135/85 mmHg) with esaxerenone was high (60.8%) and was higher than that with trichlormethiazide. However, the achievement rates for target BP level in morning home BP were found to be lower than those for bedtime home and office BP in both treatment groups. In both the J-HOP and HONEST studies, the risk of stroke was higher in patients with morning home SBP ≥ 135 mmHg versus <135 mmHg [1, 2].

In addition to its potent antihypertensive effect, esaxerenone has been shown to exert UACR-lowering effects in hypertensive patients with diabetic kidney disease, similar to finerenone [19, 29–31]. The reduction of UACR and NT-proBNP was significant compared with baseline values, but was comparable between the two groups, even though the morning home SBP reduction in the esaxerenone group was superior to the trichlormethiazide group. The predominant reduction of these biomarkers may result from the relief of hemodynamic stress due to the reduction of circulating volume by these drugs in the hypertensive patients with these biomarkers almost within the normal range. Future long-term studies on hypertensive patients with higher levels of these biomarkers are needed to conclude the difference in benefit of organ protection between the two groups.

Serum K levels ≥5.5 and ≥6.0 mEq/L were observed in 2% and 0% of patients in the esaxerenone group, respectively, which is comparable to a previous report [14]. Serum K levels increased over the first 2 weeks after starting esaxerenone treatment, then gradually decreased to a comparable level to baseline by Week 12 without specific treatment to lower serum K. A possible explanation for this observed trend may be that patients in this study were those with inadequate antihypertensive response to an ARB or CCB. Because the mineralocorticoid receptor is constantly activated in such patients, it is assumed that renal (urinary) reabsorption of sodium (Na) is enhanced and, conversely, K excretion is induced. When esaxerenone, an MRB, is administered in such patients, Na excretion is increased and K excretion is suppressed, thereby improving Na homeostasis. This effect reaches a steady state 2–4 weeks after esaxerenone administration. In terms of homeostasis, once Na homeostasis reaches normalization (steady state), K homeostasis will also normalize (i.e., serum K levels will return to normal).

The frequencies of serum K level <3.5 mEq/L were higher in the trichlormethiazide group than esaxerenone group (11.5% versus 3.1%). Serum K levels decreased over the first 2 weeks in the trichlormethiazide group, then continued to slightly decrease up to Week 12. Adverse events of hypokalemia were more frequent in this study (the trichlormethiazide group) compared with the DIME study (1.3% versus 0.4%) [32], which may be due to differences in study designs: the DIME study evaluated hypokalemia that persists after addition of K-retaining agents or K supplementation [32], whereas concomitant use of these agents was prohibited in the present study. Trichloromethiazide had a higher incidence of hyperuricemia than esaxerenone (3.7% and 1.3%, respectively) in the present study, which is consistent with the DIME study in which diuretics significantly increased UA [32]. No notable effects on other metabolic biomarkers, such as blood glucose and lipids, were observed, and these were similar in both groups. However, this study had a short 12-week study period, and a longer study period is needed to assess metabolic safety. The complication rate of hyperuricemia at baseline was approximately 15% in both groups; no patient developed gout during the study period, although UA levels were increased in the trichlormethiazide group. Given the safety results from the EXCITE-HT study, esaxerenone had no new safety concerns, and can be used safely if dose adjustments are made according to the package insert and serum K levels are regularly monitored.

MRBs, including esaxerenone, are considered effective for treatment-resistant hypertension according to the 2019 JSH guidelines [5], but are positioned as fourth-line in the list of treatments. Although previous clinical studies have demonstrated a reliable antihypertensive effect when esaxerenone is administered as second-line treatment [17–23], these studies did not compare esaxerenone with any of the five major first-line antihypertensive agents [5]. The antihypertensive effect, UACR improvement, and safety profile of esaxerenone were non-inferior to those of trichlormethiazide, which is one of the first-line agents. We hope that the EXCITE-HT study will provide new antihypertensive strategies that include treatment with esaxerenone for patients with uncontrolled hypertension.

This study has some strengths, including the large sample size and multicenter study design. Another strength of this study is that it may be generalizable to a wide range of hypertensive patients. In real-world clinical settings, ARBs or CCBs are typically administered as first-line antihypertensive agents. However, controlling BP with one ARB or CCB is often difficult.

This study has some limitations, including the open-label design and relatively short-term duration. In a longer-term study, the intergroup difference in the incidence of hypokalemia and hyperuricemia between groups may increase. Second, this study was designed to compare the usefulness of esaxerenone versus a diuretic as a second concomitant antihypertensive agent, with concomitant use of a third antihypertensive agent during the study period being prohibited. Therefore, the achievement of target morning home SBP/DBP level <125/75 mmHg was lower (esaxerenone group, 18.1%; thiazide group, 12.2%) than that of target morning home SBP/DBP level <135/85 mmHg. Long-term (10-year) prognosis is better when a target home SBP of <125 mmHg is achieved compared with ≥135 mmHg [33]. In real-world clinical practice, combination with a third agent is considered in cases of inadequate BP control, and the intergroup difference may increase in a longer-term study. Additionally, nocturnal BP was not evaluated. When morning home SBP/DBP was controlled to <135/85 mmHg, 72% of patients had better control of nocturnal SBP/DBP (<120/70 mmHg) [34]. Previous studies have also shown that esaxerenone significantly controls not only morning home BP but also nocturnal BP [18, 20, 35].

## Conclusion

EXCITE-HT is the first randomized clinical study to demonstrate that esaxerenone was non-inferior to trichlormethiazide in its antihypertensive effects based on morning home BP. Esaxerenone had a good efficacy and safety profile when administered as a second-line antihypertensive agent to patients with uncontrolled essential hypertension with one ARB or CCB.

## Supplementary information

Supplementary Materials

## References

[1] Kario K, Saito I, Kushiro T, Teramukai S, Tomono Y, Okuda Y, et al. Morning home blood pressure is a strong predictor of coronary artery disease: the HONEST Study. J Am Coll Cardiol. 2016;67:1519–27. 27150682 10.1016/j.jacc.2016.01.037

[2] Hoshide S, Yano Y, Haimoto H, Yamagiwa K, Uchiba K, Nagasaka S, et al. Morning and evening home blood pressure and risks of incident stroke and coronary artery disease in the Japanese general practice population: the Japan Morning Surge-Home Blood Pressure Study. Hypertension. 2016;68:54–61. 27160200 10.1161/HYPERTENSIONAHA.116.07201

[3] Unger T, Borghi C, Charchar F, Khan NA, Poulter NR, Prabhakaran D, et al. 2020 International Society of Hypertension Global Hypertension Practice Guidelines. Hypertension. 2020;75:1334–57. 32370572 10.1161/HYPERTENSIONAHA.120.15026

[4] Mancia G, Kreutz R, Brunström M, Burnier M, Grassi G, Januszewicz A, et al. 2023 ESH Guidelines for the management of arterial hypertension The Task Force for the management of arterial hypertension of the European Society of Hypertension: endorsed by the International Society of Hypertension (ISH) and the European Renal Association (ERA). J Hypertens. 2023;41:1874–2071. 37345492 10.1097/HJH.0000000000003480

[5] Umemura S, Arima H, Arima S, Asayama K, Dohi Y, Hirooka Y, et al. The Japanese Society of Hypertension guidelines for the management of hypertension (JSH 2019). Hypertens Res. 2019;42:1235–481. 31375757 10.1038/s41440-019-0284-9

[6] Arnett DK, Blumenthal RS, Albert MA, Buroker AB, Goldberger ZD, Hahn EJ, et al. 2019 ACC/AHA Guideline on the Primary Prevention of Cardiovascular Disease: a report of the American College of Cardiology/American Heart Association Task Force on Clinical Practice Guidelines. Circulation. 2019;140:e596–646. 30879355 10.1161/CIR.0000000000000678 PMC7734661

[7] Kario K, Tomitani N, Nishizawa M, Harada N, Kanegae H, Hoshide S. Concept, study design, and baseline blood pressure control status of the nationwide prospective HI-JAMP study using multisensor ABPM. Hypertens Res. 2023;46:357–67. 36380199 10.1038/s41440-022-01087-9

[8] Kario K, Wang JG. Could 130/80 mm Hg be adopted as the diagnostic threshold and management goal of hypertension in consideration of the characteristics of Asian populations? Hypertension. 2018;71:979–84. 29686008 10.1161/HYPERTENSIONAHA.118.11203

[9] Shibata S. Hypertension paradox in Japan: the road ahead. Hypertens Res. 2023;46:2497–9. 37644180 10.1038/s41440-023-01414-8

[10] Schwarz U. The hypertension paradox. N Engl J Med. 2009;361:2195–6. author reply 2196–7 19940307 10.1056/NEJMc0908990

[11] Arai K, Homma T, Morikawa Y, Ubukata N, Tsuruoka H, Aoki K, et al. Pharmacological profile of CS-3150, a novel, highly potent and selective non-steroidal mineralocorticoid receptor antagonist. Eur J Pharm. 2015;761:226–34. 10.1016/j.ejphar.2015.06.015 26073023

[12] Janković SM, Janković SV. Clinical pharmacokinetics and pharmacodynamics of esaxerenone, a novel mineralocorticoid receptor antagonist: a review. Eur J Drug Metab Pharmacokinet. 2022;47:291–308. 35190999 10.1007/s13318-022-00760-1

[13] Bakris GL, Agarwal R, Anker SD, Pitt B, Ruilope LM, Rossing P, et al. Effect of finerenone on chronic kidney disease outcomes in type 2 diabetes. N Engl J Med. 2020;383:2219–29. 33264825 10.1056/NEJMoa2025845

[14] Rakugi H, Yamakawa S, Sugimoto K. Management of hyperkalemia during treatment with mineralocorticoid receptor blockers: findings from esaxerenone. Hypertens Res. 2021;44:371–85. 33214722 10.1038/s41440-020-00569-y PMC8019656

[15] Ito S, Itoh H, Rakugi H, Okuda Y, Yoshimura M, Yamakawa S. Double-blind randomized phase 3 study comparing esaxerenone (CS-3150) and eplerenone in patients with essential hypertension (ESAX-HTN Study). Hypertension. 2020;75:51–8. 31786983 10.1161/HYPERTENSIONAHA.119.13569

[16] Williams B, MacDonald TM, Morant S, Webb DJ, Sever P, McInnes G, et al. Spironolactone versus placebo, bisoprolol, and doxazosin to determine the optimal treatment for drug-resistant hypertension (PATHWAY-2): a randomised, double-blind, crossover trial. Lancet. 2015;386:2059–68. 26414968 10.1016/S0140-6736(15)00257-3 PMC4655321

[17] Rakugi H, Ito S, Itoh H, Okuda Y, Yamakawa S. Long-term phase 3 study of esaxerenone as mono or combination therapy with other antihypertensive drugs in patients with essential hypertension. Hypertens Res. 2019;42:1932–41. 31554937 10.1038/s41440-019-0314-7 PMC8076031

[18] Kario K, Ito S, Itoh H, Rakugi H, Okuda Y, Yamakawa S. Effect of esaxerenone on nocturnal blood pressure and natriuretic peptide in different dipping phenotypes. Hypertens Res. 2022;45:97–105. 34650195 10.1038/s41440-021-00756-5 PMC8668432

[19] Uchida HA, Nakajima H, Hashimoto M, Nakamura A, Nunoue T, Murakami K, et al. Efficacy and safety of esaxerenone in hypertensive patients with diabetic kidney disease: a multicenter, open-label, prospective study. Adv Ther. 2022;39:5158–75. 36070133 10.1007/s12325-022-02294-z PMC9449923

[20] Kario K, Nishizawa M, Kato M, Ishii H, Uchiyama K, Nagai M, et al. Nighttime home blood pressure lowering effect of esaxerenone in patients with uncontrolled nocturnal hypertension: the EARLY-NH study. Hypertens Res. 2023;46:1782–94. 37173430 10.1038/s41440-023-01292-0 PMC10319630

[21] Motoki H, Inobe Y, Fukui T, Iwasaki A, Hiramitsu S, Koyama S, et al. Efficacy and safety of esaxerenone in hypertensive patients with diabetes mellitus undergoing treatment with sodium-glucose cotransporter 2 inhibitors (EAGLE-DH). Adv Ther. 2023;40:5055–75. 37733211 10.1007/s12325-023-02633-8 PMC10567833

[22] Katsuya T, Inobe Y, Uchiyama K, Nishikawa T, Hirano K, Kato M, et al. Exploratory study on the relationship between urinary sodium/potassium ratio, salt intake, and the antihypertensive effect of esaxerenone: the ENaK Study. Hypertens Res. 2024;47:835–48. 38212366 10.1038/s41440-023-01519-0 PMC10994843

[23] Yamamoto E, Usuku H, Sueta D, Suzuki S, Nakamura T, Matsui K, et al. Efficacy and safety of esaxerenone in hypertensive patients with left ventricular hypertrophy (ESES-LVH) study: a multicenter, open-label, prospective, interventional study. Adv Ther. 2024;41:1284–303. 38310194 10.1007/s12325-024-02780-6 PMC10879332

[24] Kario K, Ohishi M, Katsuya T, Taguchi T, Tanabe A, Sugimoto K, et al. Rationale and design of a multicenter randomized study comparing the efficacy and safety of esaxerenone versus trichlormethiazide in patients with uncontrolled essential hypertension: EXCITE-HT study. J Clin Hypertens. 2023;25:861–7. 10.1111/jch.14705 PMC10497029 37551054

[25] Esaxerenone (Minnebro) tablets [Japanese package insert]. Tokyo, Japan: Daiichi Sankyo Co., Ltd.; 2022. https://pins.japic.or.jp/pdf/newPINS/00070243.pdf [In Japanese]. Accessed March 28, 2024.

[26] Trichlormethiazide (Fluitran) tablets [Japanese package insert]. Osaka, Japan: Shionogi & Co., Ltd.; 2019. https://pins.japic.or.jp/pdf/newPINS/00056852.pdf [In Japanese]. Accessed March 28, 2024.

[27] Kario K, Sakima A, Ohya Y. STEP to estimate cardiovascular events by home blood pressure in the era of digital hypertension. Hypertens Res. 2022;45:11–4. 34657134 10.1038/s41440-021-00764-5

[28] Kario K, Tomitani N, Kanegae H, Ishii H, Uchiyama K, Yamagiwa K, et al. Comparative effects of an angiotensin II receptor blocker (ARB)/diuretic vs. ARB/calcium-channel blocker combination on uncontrolled nocturnal hypertension evaluated by information and communication technology-based nocturnal home blood pressure monitoring - the NOCTURNE Study. Circ J. 2017;81:948–57. 28321001 10.1253/circj.CJ-17-0109

[29] Itoh H, Ito S, Rakugi H, Okuda Y, Nishioka S. Efficacy and safety of dosage-escalation of low-dosage esaxerenone added to a RAS inhibitor in hypertensive patients with type 2 diabetes and albuminuria: a single-arm, open-label study. Hypertens Res. 2019;42:1572–81. 31239535 10.1038/s41440-019-0270-2 PMC8075891

[30] Ito S, Itoh H, Rakugi H, Okuda Y, Iijima S. Antihypertensive effects and safety of esaxerenone in patients with moderate kidney dysfunction. Hypertens Res. 2021;44:489–97. 33323991 10.1038/s41440-020-00585-y PMC8099724

[31] Ito S, Kashihara N, Shikata K, Nangaku M, Wada T, Okuda Y, et al. Esaxerenone (CS-3150) in patients with type 2 diabetes and microalbuminuria (ESAX-DN): phase 3 randomized controlled clinical trial. Clin J Am Soc Nephrol. 2020;15:1715–27. 33239409 10.2215/CJN.06870520 PMC7769030

[32] Ueda S, Morimoto T, Ando S, Takishita S, Kawano Y, Shimamoto K, et al. A randomised controlled trial for the evaluation of risk for type 2 diabetes in hypertensive patients receiving thiazide diuretics: Diuretics In the Management of Essential hypertension (DIME) study. BMJ Open. 2014;4:e004576. 25031188 10.1136/bmjopen-2013-004576 PMC4120409

[33] Kario K, Okawara Y, Kanegae H, Hoshide S. Potential long-term benefit of home systolic blood pressure below 125 mm hg for cardiovascular risk reduction: the J-HOP study extended. Hypertension. 2024;81:282–90. 38073531 10.1161/HYPERTENSIONAHA.123.22122

[34] Kario K, Tomitani N, Hoshide S, Nishizawa M, Yoshida T, Kabutoya T, et al. Different home blood pressure thresholds to predict perfect 24-hour ambulatory blood pressure control in treated hypertension based on an “all-in-one” device. Hypertension. 2023;80:2464–72. 37671575 10.1161/HYPERTENSIONAHA.123.21578

[35] Kario K, Ito S, Itoh H, Rakugi H, Okuda Y, Yoshimura M, et al. Effect of the nonsteroidal mineralocorticoid receptor blocker, esaxerenone, on nocturnal hypertension: a post hoc analysis of the ESAX-HTN study. Am J Hypertens. 2021;34:540–51. 33165570 10.1093/ajh/hpaa155 PMC8140658

