# Supplementary material for: Home blood pressure-lowering effect of a non-steroidal mineralocorticoid receptor blocker, esaxerenone, versus trichlormethiazide for uncontrolled hypertension: the EXCITE-HT randomized controlled study
Source: Hypertens Res. 2024 Jul 23;47(9):2435–46. doi: 10.1038/s41440-024-01762-z (PMC11374750; doi:10.1038/s41440-024-01762-z)
Supplement: Supplementary file 1 — Supplementary Materials [file 41440_2024_1762_MOESM1_ESM.docx]

**Supplementary Materials**

**Supplementary Table 1.** Participating institutions and representative physicians

| **Institutions*** | **Representative physicians** |
| --- | --- |
| Division of Cardiovascular Medicine, Department of Medicine, Jichi Medical University School of Medicine | Kazuomi Kario (Principal Investigator) |
| Akaicho Clinic | Hidenori Ishida |
| Asano Clinic | Hideto Ishii |
| Ebe Clinic | Yusuke Ebe |
| Hashimoto Kidney Clinic | Masami Hashimoto |
| Hatta Medical Clinic | Tsuguru Hatta |
| Higashirinkan Kaneshiro Medical Clinic | Mizuki Kaneshiro |
| Hirano Clinic | Kunio Hirano |
| Hiratsuka Gastroenterological Hospital | Daisuke Sakaguchi |
| Igarashi Internal Medicine Surgery Clinic | Hironori Kaneko |
| Inobe Funai Clinic | Yoshito Inobe |
| Inomata Clinic | Masahiko Inomata |
| Inoue Internal Medicine Clinic | Masanori Inoue |
| Ishikawa Prefectural Central Hospital | Toshihiko Yasuda |
| Itabashi Diabetes and Dermatology Medical Clinic | Naoki Itabashi |
| Iwase General Hospital | Hiroshi Ohtani |
| Jinaido Clinic | Shunichi Kobayashi |
| Kajiyama Clinic | Shizuo Kajiyama |
| Kato Clinic of Internal Medicine | Mitsutoshi Kato |
| Katsuya Clinic | Tomohiro Katsuya |
| Kenkokan Suzuki Clinic of Internal Medicine | Kazuo Suzuki |
| Kikuma Clinic | Kenji Yamauchi |
| Kodaira Hospital | Makoto Kodaira |
| Kotani Diabetes Clinic | Kei Kotani |
| Koukan Clinic, Nippon Koukan Hospital | Takashi Udagawa |
| Kyoundo Hospital | Katsuaki Okubo |
| Maebashi Hirosegawa Clinic | Shintaro Yano |
| Matsumoto Clinic | Keitoku Matsumoto |
| Medical Corporation Association Koukeikai Sugiura Clinic | Toshiyuki Sugiura |
| Medical Corporation Kyoujinkai Komatsu Hospital | Soichi Kurioka |
| Medical Corporation Shirayurikai Swing Nozaki Clinic | Minoru Nozaki |
| Minamino Cardiovascular Hospital | Yoshiki Hata |
| Miyamoto Internal Medicine Clinic | Takahide Miyamoto |
| Murakami Clinic | Kazuharu Murakami |
| Nakatani Hospital | Yuji Nakatani |
| Nakayama Clinic | Mikihiro Nakayama |
| Nakayamadera Imai Clinic | Enyu Imai |
| Nerima General Hospital | Kashima Ito |
| Nihonbashi Sakura Clinic | Kumie Ito |
| Nishikawa Clinic | Tetsuo Nishikawa |
| Olive Takamatsu Medical Clinic | Toshiki Fukui |
| Primula Clinic | Noriko Nakamura |
| Saino Clinic | Naomasa Miyamoto |
| Sanin Rosai Hospital | Shuichi Osaki |
| Sato Hospital | Tatsuyuki Sato |
| Shimokitazawa Tomo Clinic | Tomofumi Murakami |
| Tohno Chuo Clinic | Hiroyuki Ohbayashi |
| Tokyo Center Clinic | Hirotaka Nagashima |
| Tsuruma Kaneshiro Medical Clinic | Taro Asakura |
| Uchiyama Clinic | Kazuaki Uchiyama |
| Washiya Hospital | Toshimitsu Kitajima |
| Yamada Clinic | Hiroyuki Yamada |
| Yamagishi Clinic Sagamiono | Takahiro Yamagishi |
| Yamaichi Bldg. Medical Clinic | Toru Arino |

*The institution of the Principal Investigator is listed first, with all other participating institutions listed alphabetically.

**Supplementary Table 2.** Baseline patient characteristics (per protocol set)

| **Characteristics** | **Esaxerenone**  ***n* = 275** | **Trichlormethiazide**  ***n* = 290** |
| --- | --- | --- |
| Sex, male | 143 (52.0) | 160 (55.2) |
| Age, years | 65.1 ± 11.6 | 64.7 ± 12.1 |
| Weight, kg | 65.54 ± 14.57 | 66.63 ± 13.90 |
| Body mass index, kg/m^2^ | 25.27 ± 4.16 | 25.53 ± 4.20 |
| Morning home SBP, mmHg | 140.7 ± 15.2 | 139.4 ± 13.1 |
| Morning home DBP, mmHg | 87.1 ± 9.8 | 86.6 ± 9.4 |
| Bedtime home SBP, mmHg | 135.4 ± 16.0 | 134.4 ± 14.0 |
| Bedtime home DBP, mmHg | 81.8 ± 10.6 | 81.3 ± 10.8 |
| Office SBP, mmHg | 143.6 ± 16.6 | 142.7 ± 15.1 |
| Office DBP, mmHg | 83.5 ± 11.7 | 83.4 ± 12.2 |
| NT-proBNP, pg/mL | 110.77 ± 317.71  51.00 (0.5, 4229.0) | 83.84 ± 147.38  46.00 (0.5, 1763.0) |
| <55 | 120 (43.6) | 140 (48.3) |
| 55 to <125 | 58 (21.1) | 72 (24.8) |
| ≥125 | 45 (16.4) | 41 (14.1) |
| UACR, mg/gCr | 117.36 ± 493.12  14.60 (2.0, 6600.0) | 101.35 ± 424.41  17.95 (1.7, 6430.0) |
| <30 | 178 (64.7) | 187 (64.5) |
| 30 to <300 | 79 (28.7) | 83 (28.6) |
| ≥300 | 18 (6.5) | 20 (6.9) |
| Serum K, mEq/L | 4.21 ± 0.35 | 4.21 ± 0.32 |
| Uric acid, mg/dL | 5.41 ± 1.27 | 5.40 ± 1.20 |
| eGFRcreat, mL/min/1.73 m^2^ | 71.77 ± 15.90 | 72.13 ± 17.09 |
| Duration of hypertension, years | 5.29 ± 5.06 | 5.46 ± 5.03 |
| Complication | 266 (96.7) | 276 (95.2) |
| T2DM | 106 (38.5) | 115 (39.7) |
| Dyslipidemia | 176 (64.0) | 171 (59.0) |
| Hyperuricemia | 43 (15.6) | 44 (15.2) |
| Heart failure | 19 (6.9) | 16 (5.5) |
| Dose of esaxerenone at baseline (initial dose), mg |  |  |
| 1.25 | 104 (37.8) | - |
| 2.5 | 171 (62.2) | - |
| Dose of esaxerenone at EOT (last dose), mg |  |  |
| 1.25 | 55 (20.0) | - |
| 2.5 | 165 (60.0) | - |
| 5 | 55 (20.0) | - |
| Dose of trichlormethiazide at baseline (initial dose), mg |  |  |
| 0.25 | - | 4 (1.4) |
| 0.5 | - | 17 (5.9) |
| 1 | - | 262 (90.3) |
| 2 | - | 7 (2.4) |
| Dose of trichlormethiazide at EOT (last dose), mg |  |  |
| 0.25 | - | 2 (0.7) |
| 0.5 | - | 18 (6.2) |
| 1 | - | 244 (84.1) |
| > 1 to ≤2 | - | 24 (8.3) |
| ≥3 | - | 2 (0.7) |
| Basal antihypertensive agent |  |  |
| ARB | 108 (39.3) | 116 (40.0) |
| CCB | 167 (60.7) | 174 (60.0) |

Data are *n* (%), mean ± standard deviation, or median (minimum, maximum).

*ARB* angiotensin receptor blocker, *CCB* calcium channel blocker, *DBP* diastolic blood pressure, *eGFRcreat* creatinine-based estimated glomerular filtration rate, *EOT* end of treatment, *K* potassium, *NT-proBNP* N-terminal pro-brain natriuretic peptide, *SBP* systolic blood pressure, *T2DM* type 2 diabetes mellitus, *UACR* urinary albumin-to-creatinine ratio.

**Supplementary Table 3.** Change from baseline in BP (full analysis set)

| **BP** | **Esaxerenone** | | | **Trichlormethiazide** | | |
| --- | --- | --- | --- | --- | --- | --- |
|  | ***n*** | **SBP, mmHg** | **DBP, mmHg** | ***n*** | **SBP, mmHg** | **DBP, mmHg** |
| Morning home BP |  |  |  |  |  |  |
| Baseline | 295 | 140.1 ± 15.0 | 86.8 ± 9.6 | 290 | 139.4 ± 13.1 | 86.6 ± 9.4 |
| Week 2 | 295 | 133.5 ± 14.7 | 83.7 ± 9.3 | 290 | 133.3 ± 12.8 | 83.6 ± 9.4 |
| Change from baseline | 295 | −6.6 ± 7.6* | −3.1 ± 4.3* | 290 | −6.1 ± 8.5* | −3.0 ± 4.7* |
| Week 4 | 295 | 131.4 ± 14.8 | 82.4 ± 9.4 | 290 | 132.5 ± 13.2 | 82.9 ± 9.3 |
| Change from baseline | 295 | −8.6 ± 8.7* | −4.4 ± 4.8* | 290 | −7.0 ± 8.8* | −3.7 ± 4.6* |
| Week 6 | 291 | 130.4 ± 14.9 | 81.6 ± 9.4 | 289 | 131.5 ± 13.0 | 82.1 ± 9.2 |
| Change from baseline | 291 | −9.7 ± 8.9* | −5.3 ± 5.0* | 289 | −7.9 ± 9.3* | −4.4 ± 5.1* |
| Week 8 | 289 | 130.3 ± 14.4 | 81.7 ± 9.4 | 288 | 130.8 ± 13.0 | 81.5 ± 9.4 |
| Change from baseline | 289 | −9.8 ± 9.0* | −5.1 ± 5.4* | 288 | −8.7 ± 9.8* | −5.0 ± 5.2* |
| Week 10 | 286 | 128.4 ± 13.7 | 80.5 ± 9.4 | 285 | 130.3 ± 12.5 | 81.4 ± 9.6 |
| Change from baseline | 286 | −11.6 ± 10.4* | −6.3 ± 5.7* | 285 | −9.1 ± 9.3* | −5.1 ± 5.0* |
| Week 12 | 283 | 128.0 ± 13.0 | 80.5 ± 9.4 | 285 | 130.1 ± 12.1 | 81.0 ± 9.6 |
| Change from baseline | 283 | −12.0 ± 10.4* | −6.4 ± 5.7* | 285 | −9.4 ± 9.1* | −5.5 ± 5.3* |
| LS mean change from baseline [95% CI] | 283 | −12.2 [−13.2, −11.2] | −6.5 [−7.1, −6.0] | 285 | −9.8 [−10.8, −8.8] | −5.8 [−6.4, −5.2] |
| Difference in LS mean change from baseline [95% CI] |  | −2.4 [−3.7, −1.0] | −0.8 [−1.6, 0.1] |  |  |  |
| EOT | 294 | 128.1 ± 13.2 | 80.5 ± 9.3 | 290 | 129.9 ± 12.2 | 81.0 ± 9.5 |
| Change from baseline | 294 | −12.0 ± 10.6* | −6.3 ± 5.8* | 290 | −9.5 ± 9.2* | −5.6 ± 5.3* |
| LS mean change from baseline [95% CI] | 294 | −12.2 [−13.2, −11.2] | −6.5 [−7.0, −5.9] | 290 | −10.0 [−11.0, −9.0] | −5.9 [−6.4, −5.3] |
| Difference in LS mean change from baseline [95% CI] |  | −2.2 [−3.6, −0.8] | −0.6 [−1.4, 0.2] |  |  |  |
| Bedtime home BP |  |  |  |  |  |  |
| Baseline | 281 | 134.7 ± 15.8 | 81.5 ± 10.5 | 282 | 134.4 ±14.0 | 81.3 ± 10.8 |
| Week 2 | 289 | 128.5 ± 15.5 | 78.9 ± 10.3 | 288 | 129.0 ± 13.2 | 78.4 ± 10.2 |
| Change from baseline | 281 | −6.4 ± 8.1* | −2.6 ± 4.5* | 282 | −5.6 ± 8.0* | −2.8 ± 4.9* |
| Week 4 | 292 | 126.7 ± 15.1 | 77.5 ± 10.1 | 288 | 128.0 ± 13.0 | 77.8 ± 9.8 |
| Change from baseline | 281 | −8.4 ± 9.0* | −4.2 ± 5.2* | 282 | −6.5 ± 8.9* | −3.5 ± 5.6* |
| Week 6 | 288 | 125.7 ± 14.7 | 76.9 ± 10.1 | 287 | 126.7 ± 12.8 | 77.0 ± 9.8 |
| Change from baseline | 277 | −9.3 ± 9.6* | −4.8 ± 5.5* | 281 | −7.8 ± 9.2* | −4.2 ± 5.9* |
| Week 8 | 286 | 125.0 ± 14.5 | 76.3 ± 10.1 | 286 | 126.3 ± 12.9 | 76.6 ± 9.9 |
| Change from baseline | 275 | −9.9 ± 9.5* | −5.3 ± 5.7* | 280 | −8.3 ± 9.8* | −4.7 ± 5.9* |
| Week 10 | 283 | 123.9 ± 13.2 | 75.9 ± 9.6 | 283 | 125.7 ± 13.4 | 75.8 ± 10.3 |
| Change from baseline | 272 | −11.1 ± 10.8* | −5.9 ± 6.2* | 277 | −8.8 ± 10.4* | −5.4 ± 6.2* |
| Week 12 | 280 | 123.8 ± 13.3 | 75.4 ± 9.9 | 283 | 125.8 ± 13.5 | 76.0 ± 10.0 |
| Change from baseline | 269 | −11.3 ± 10.5* | −6.4 ± 6.1* | 277 | −8.7 ± 10.1* | −5.2 ± 6.4* |
| EOT | 291 | 123.8 ± 13.5 | 75.4 ± 9.8 | 288 | 125.6 ± 13.5 | 75.9 ± 10.0 |
| Change from baseline | 280 | −11.1 ± 10.5* | −6.2 ± 6.1* | 282 | −8.9 ± 10.1* | −5.3 ± 6.4* |
| Office BP |  |  |  |  |  |  |
| Baseline | 295 | 143.8 ± 16.5 | 83.5 ± 11.6 | 290 | 142.7 ± 15.1 | 83.4 ± 12.2 |
| Week 2 | 294 | 135.9 ± 16.7 | 80.2 ± 11.8 | 290 | 135.8 ± 15.2 | 80.4 ± 12.0 |
| Change from baseline | 294 | −7.9 ± 12.3* | −3.2 ± 7.8* | 290 | −6.9 ± 12.6* | −3.0 ± 8.3* |
| Week 4 | 295 | 135.3 ± 15.9 | 79.3 ± 11.0 | 290 | 135.0 ± 14.6 | 79.7 ± 11.4 |
| Change from baseline | 295 | −8.5 ± 12.7* | −4.2 ± 8.2* | 290 | −7.8 ± 12.7* | −3.8 ± 8.4* |
| Week 8 | 287 | 132.5 ± 16.6 | 78.1 ± 11.5 | 286 | 134.3 ± 15.4 | 78.7 ± 12.4 |
| Change from baseline | 287 | −11.2 ± 12.8* | −5.3 ± 8.2* | 286 | −8.4 ± 12.8* | −4.6 ± 8.9* |
| Week 12 | 283 | 130.7 ± 16.0 | 77.0 ± 11.5 | 284 | 133.0 ± 14.6 | 78.5 ± 12.0 |
| Change from baseline | 283 | −13.1 ± 13.1* | −6.4 ± 8.8* | 284 | −9.5 ± 12.8* | −4.9 ± 8.7* |
| EOT | 294 | 130.7 ± 15.8 | 77.0 ± 11.4 | 290 | 133.1 ± 14.8 | 78.5 ± 12.1 |
| Change from baseline | 294 | −13.0 ± 13.1* | −6.4 ± 8.8* | 290 | −9.6 ± 12.7* | −4.9 ± 8.6* |

**P* < 0.001 versus baseline, paired *t*-test.

LS mean change were calculated for morning home BP at Week 12 and EOT.

LS mean change and 95% CIs were calculated using the analysis of covariance model, with morning home SBP/DBP change from baseline as the objective variable; treatment group as the explanatory variable; and baseline BP, baseline antihypertensive medication, and baseline age as covariates.

*P*-values were not calculated for changes from baseline to Week 4.

*BP* blood pressure, *CI* confidence interval; *DBP* diastolic blood pressure, *EOT* end of treatment, *LS* least squares, *SBP* systolic blood pressure, *SD* standard deviation.

**Supplementary Table 4.** Change from baseline in BP (per protocol set)

| **BP** | **Esaxerenone** | | | **Trichlormethiazide** | | |
| --- | --- | --- | --- | --- | --- | --- |
|  | ***n*** | **SBP, mmHg** | **DBP, mmHg** | ***n*** | **SBP, mmHg** | **DBP, mmHg** |
| Morning home BP |  |  |  |  |  |  |
| Baseline | 275 | 140.7 ± 15.2 | 87.1 ± 9.8 | 290 | 139.4 ± 13.1 | 86.6 ± 9.4 |
| Week 2 | 275 | 134.1 ± 14.7 | 84.0 ± 9.4 | 290 | 133.3 ± 12.8 | 83.6 ± 9.4 |
| Change from baseline | 275 | −6.6 ± 7.7* | −3.1 ± 4.3* | 290 | −6.1 ± 8.5* | −3.0 ± 4.7* |
| Week 4 | 275 | 132.0 ± 14.9 | 82.6 ± 9.5 | 290 | 132.5 ± 13.2 | 82.9 ± 9.3 |
| Change from baseline | 275 | −8.7 ± 8.8* | −4.5 ± 4.9* | 290 | −7.0 ± 8.8* | −3.7 ± 4.6* |
| Week 6 | 272 | 130.8 ± 15.0 | 81.8 ± 9.4 | 289 | 131.5 ± 13.0 | 82.1 ± 9.2 |
| Change from baseline | 272 | −9.8 ± 8.9* | −5.3 ± 5.0* | 289 | −7.9 ± 9.3* | −4.4 ± 5.1* |
| Week 8 | 270 | 130.6 ± 14.6 | 81.8 ± 9.6 | 288 | 130.8 ± 13.0 | 81.5 ± 9.4 |
| Change from baseline | 270 | −9.9 ± 9.0* | −5.2 ± 5.3* | 288 | −8.7 ± 9.8* | −5.0 ± 5.2* |
| Week 10 | 267 | 128.6 ± 13.8 | 80.6 ± 9.5 | 285 | 130.3 ± 12.5 | 81.4 ± 9.6 |
| Change from baseline | 267 | −11.9 ± 10.3* | −6.4 ± 5.6* | 285 | −9.1 ± 9.3* | −5.1 ± 5.0* |
| Week 12 | 264 | 128.2 ± 13.1 | 80.6 ± 9.6 | 285 | 130.1 ± 12.1 | 81.0 ± 9.6 |
| Change from baseline | 264 | −12.3 ± 10.4* | −6.5 ± 5.6* | 285 | −9.4 ± 9.1* | −5.5 ± 5.3* |
| LS mean change from baseline [95% CI] | 264 | −12.5 [−13.5, −11.5] | −6.7 [−7.3, −6.1] | 285 | −9.9 [−10.9, −8.9] | −5.8 [−6.4, −5.2] |
| Difference in LS mean change from baseline [95% CI] |  | −2.6 [−4.0, −1.2] | −0.9 [−1.7, −0.1] |  |  |  |
| EOT | 274 | 128.4 ± 13.2 | 80.6 ± 9.5 | 290 | 129.9 ± 12.2 | 81.0 ± 9.5 |
| Change from baseline | 274 | −12.3 ± 10.5* | −6.4 ± 5.7* | 290 | −9.5 ± 9.2* | −5.6 ± 5.3* |
| LS mean change from baseline [95% CI] | 274 | −12.5 [−13.5, −11.5] | −6.6 [−7.2, −6.0] | 290 | −10.1 [−11.1, −9.1] | −5.9 [−6.5, −5.3] |
| Difference in LS mean change from baseline [95% CI] |  | −2.4 [−3.8, −1.0] | −0.7 [−1.5, 0.1] |  |  |  |
| Bedtime home BP |  |  |  |  |  |  |
| Baseline | 261 | 135.4 ± 16.0 | 81.8 ± 10.6 | 282 | 134.4 ± 14.0 | 81.3 ± 10.8 |
| Week 2 | 269 | 129.3 ± 15.6 | 79.3 ± 10.3 | 288 | 129.0 ± 13.2 | 78.4 ± 10.2 |
| Change from baseline | 261 | −6.3 ± 8.1* | −2.5 ± 4.5* | 282 | −5.6 ± 8.0* | −2.8 ± 4.9* |
| Week 4 | 272 | 127.2 ± 15.4 | 77.7 ± 10.4 | 288 | 128.0 ± 13.0 | 77.8 ± 9.8 |
| Change from baseline | 261 | −8.6 ± 9.1* | −4.3 ± 5.3* | 282 | −6.5 ± 8.9* | −3.5 ± 5.6* |
| Week 6 | 269 | 126.1 ± 15.0 | 77.1 ± 10.2 | 287 | 126.7 ± 12.8 | 77.0 ± 9.8 |
| Change from baseline | 258 | −9.5 ± 9.6* | −4.8 ± 5.5* | 281 | −7.8 ± 9.2* | −4.2 ± 5.9* |
| Week 8 | 267 | 125.4 ± 14.7 | 76.4 ± 10.3 | 286 | 126.3 ± 12.9 | 76.6 ± 9.9 |
| Change from baseline | 256 | −10.1 ± 9.3* | −5.4 ± 5.5* | 280 | −8.3 ± 9.8* | −4.7 ± 5.9* |
| Week 10 | 264 | 124.1 ± 13.4 | 76.0 ± 9.7 | 283 | 125.7 ± 13.4 | 75.8 ± 10.3 |
| Change from baseline | 253 | −11.5 ± 10.4* | −6.1 ± 5.9* | 277 | −8.8 ± 10.4* | −5.4 ± 6.2* |
| Week 12 | 261 | 124.1 ± 13.5 | 75.6 ± 10.0 | 283 | 125.8 ± 13.5 | 76.0 ± 10.0 |
| Change from baseline | 250 | −11.7 ± 10.4* | −6.5 ± 5.9* | 277 | −8.7 ± 10.1* | −5.2 ± 6.4* |
| EOT | 271 | 124.1 ± 13.6 | 75.6 ± 9.9 | 288 | 125.6 ± 13.5 | 75.9 ± 10.0 |
| Change from baseline | 260 | −11.5 ± 10.5* | −6.3 ± 6.0* | 282 | −8.9 ± 10.1* | −5.3 ± 6.4* |
| Office BP |  |  |  |  |  |  |
| Baseline | 275 | 143.6 ± 16.6 | 83.5 ± 11.7 | 290 | 142.7 ± 15.1 | 83.4 ± 12.2 |
| Week 2 | 274 | 135.9 ± 17.0 | 80.3 ± 11.9 | 290 | 135.8 ± 15.2 | 80.4 ± 12.0 |
| Change from baseline | 274 | −7.6 ± 12.0* | −3.2 ± 7.9* | 290 | −6.9 ± 12.6* | −3.0 ± 8.3* |
| Week 4 | 275 | 135.5 ± 16.3 | 79.3 ± 11.3 | 290 | 135.0 ± 14.6 | 79.7 ± 11.4 |
| Change from baseline | 275 | −8.1 ± 12.4* | −4.1 ± 8.3* | 290 | −7.8 ± 12.7* | −3.8 ± 8.4* |
| Week 8 | 268 | 132.5 ± 16.9 | 78.0 ± 11.7 | 286 | 134.3 ± 15.4 | 78.7 ± 12.4 |
| Change from baseline | 268 | −11.0 ± 12.8* | −5.4 ± 8.3* | 286 | −8.4 ± 12.8* | −4.6 ± 8.9* |
| Week 12 | 264 | 130.8 ± 16.3 | 77.1 ± 11.7 | 284 | 133.0 ± 14.6 | 78.5 ± 12.0 |
| Change from baseline | 264 | −12.8 ± 13.0* | −6.3 ± 8.9* | 284 | −9.5 ± 12.8* | −4.9 ± 8.7* |
| EOT | 274 | 130.7 ± 16.2 | 77.0 ± 11.6 | 290 | 133.1 ± 14.8 | 78.5 ± 12.1 |
| Change from baseline | 274 | −12.7 ± 13.1* | −6.3 ± 8.9* | 290 | −9.6 ± 12.7* | −4.9 ± 8.6* |

**p*<0.001 versus baseline, paired *t*-test.

LS mean change were calculated for morning home BP at Week 12 and EOT.

LS mean change and 95% CIs were calculated using the analysis of covariance model, with morning home SBP/DBP change from baseline as the objective variable; treatment group as the explanatory variable; and baseline BP, baseline antihypertensive medication, and baseline age as covariates.

*P*-values were not calculated for changes from baseline to Week 4.

*BP* blood pressure, *CI* confidence interval; *DBP* diastolic blood pressure, *EOT* end of treatment, *LS* least squares, *SBP* systolic blood pressure, *SD* standard deviation.

**Supplementary Table 5.** Achievement rate of target BP levels at Week 12 (per protocol set)

|  | **Criteria 1**  **Home SBP/DBP of <135/85 mmHg**  **Office SBP/DBP of <140/90 mmHg** | | | | **Criteria 2**  **Home SBP/DBP of <125/75 mmHg**  **Office SBP/DBP of <130/80 mmHg** | | | |
| --- | --- | --- | --- | --- | --- | --- | --- | --- |
|  | **Esaxerenone** | | **Trichlormethiazide** | | **Esaxerenone** | | **Trichlormethiazide** | |
| **BP** | ***n*** | **Achievement rate** | ***n*** | **Achievement rate** | ***n*** | **Achievement rate** | ***n*** | **Achievement rate** |
| Morning home BP | 264 | 60.6 (54.4, 66.5) | 285 | 55.8 (49.8, 61.6) | 242 | 18.2 (13.5, 23.6) | 263 | 12.2 (8.5, 16.7) |
| Bedtime home BP | 261 | 77.0 (71.4, 82.0) | 283 | 71.0 (65.4, 76.2) | 239 | 36.8 (30.7, 43.3) | 261 | 27.6 (22.3, 33.4) |
| Office BP | 264 | 71.6 (65.7, 77.0) | 284 | 64.8 (58.9, 70.3) | 242 | 34.3 (28.3, 40.6) | 262 | 28.2 (22.9, 34.1) |

Data are % (95% CI) calculated using the Clopper–Pearson method.

Criterion 1 was analyzed in all patients, and criterion 2 was analyzed in patients who were aged <75 years, or had CKD (≥UACR 30 mg/gCr), or diabetes mellitus.

*BP* blood pressure, *CI* confidence interval, *CKD* chronic kidney disease, *DBP* diastolic blood pressure, *SBP* systolic blood pressure, *UACR* urinary albumin-to-creatinine ratio.

**Supplementary Table 6.** Change in UACR and NT-proBNP from baseline to Week 12 (full analysis set)

| **Variables** | ***n*** | **Esaxerenone** | ***n*** | **Trichlormethiazide** |
| --- | --- | --- | --- | --- |
| **UACR, mg/gCr** |  |  |  |  |
| Baseline | 295 | 116.27 ± 482.31 | 290 | 101.35 ± 424.41 |
| Week 4 | 295 | 77.86 ± 410.66 | 290 | 70.17 ± 326.86 |
| Change from baseline | 295 | −38.41 ± 180.32 | 290 | −31.19 ± 190.80 |
| Percentage change in geometric mean from baseline [95% CI] |  | −33.0  [−38.4, −27.1]* |  | −28.9  [−34.9, −22.4]* |
| Week 8 | 287 | 50.05 ± 158.84 | 286 | 55.51 ± 250.03 |
| Change from baseline | 287 | −34.96 ± 147.48 | 286 | −46.88 ± 215.79 |
| Percentage change in geometric mean from baseline [95% CI] |  | −38.3  [−43.8, −32.3]* |  | −35.7  [−41.1, −29.8]* |
| Week 12 | 283 | 52.79 ± 230.61 | 284 | 54.36 ± 259.72 |
| Change from baseline | 283 | −29.17 ± 176.76 | 284 | −48.45 ± 221.89 |
| Percentage change in geometric mean from baseline [95% CI] |  | −38.9  [−45.1, −32.0]* |  | −41.9  [−46.9, −36.4]* |
| **NT-proBNP, pg/mL** |  |  |  |  |
| Baseline | 237 | 109.49 ± 308.62 | 253 | 83.84 ± 147.38 |
| Week 12 | 220 | 77.51 ± 120.57 | 241 | 75.44 ± 120.02 |
| Change from baseline | 220 | −33.09 ± 243.79^†^ | 238 | −9.53 ± 91.34 |

**p*<0.001, ^†^*p*<0.05 versus baseline, paired *t*-test.

For UACR, *P-*values are only presented for percentage change in geometric mean from baseline.

*CI* confidence interval, *NT-proBNP* N-terminal pro-brain natriuretic peptide, *UACR* urinary albumin-to-creatinine ratio.

**Supplementary Table 7.** Change in UACR and NT-proBNP from baseline to Week 12 (per protocol set)

| **Variables** | ***n*** | **Esaxerenone** |  | **Trichlormethiazide** |
| --- | --- | --- | --- | --- |
| **UACR, mg/gCr** |  |  |  |  |
| Baseline | 275 | 117.36 ± 493.12 | 290 | 101.35 ± 424.41 |
| Week 4 | 275 | 80.03 ± 424.15 | 290 | 70.17 ± 326.86 |
| Change from baseline | 275 | −37.34 ± 179.89 | 290 | −31.19 ± 190.80 |
| Percentage change in geometric mean from baseline [95% CI] |  | −32.3  [−38.1, −26.0]* |  | −28.9  [−34.9, −22.4]* |
| Week 8 | 268 | 51.23 ± 163.33 | 286 | 55.51 ± 250.03 |
| Change from baseline | 268 | −32.34 ± 138.34 | 286 | −46.88 ± 215.79 |
| Percentage change in geometric mean from baseline [95% CI] |  | −38.1  [−43.9, −31.6]* |  | −35.7  [−41.1, −29.8]* |
| Week 12 | 264 | 53.19 ± 236.51 | 284 | 54.36 ± 259.72 |
| Change from baseline | 264 | −27.08 ± 176.16 | 284 | −48.45 ± 221.89 |
| Percentage change in geometric mean from baseline [95% CI] |  | −38.4  [−45.0, −31.1]* |  | −41.9  [−46.9, −36.4]* |
| **NT-proBNP, pg/mL** |  |  |  |  |
| Baseline | 223 | 110.77 ± 317.71 | 253 | 83.84 ± 147.38 |
| Week 12 | 207 | 77.71 ± 122.78 | 241 | 75.44 ± 120.02 |
| Change from baseline | 207 | −34.57 ± 251.16^†^ | 238 | −9.53 ± 91.34 |

**p*<0.001, ^†^*p*<0.05 versus baseline, paired *t*-test.

For UACR, *P-*values are only presented for percentage change in geometric mean from baseline.

*CI* confidence interval, *NT-proBNP* N-terminal pro-brain natriuretic peptide, *UACR* urinary albumin-to-creatinine ratio.

**Supplementary Table 8.** Change in UA, blood electrolytes, eGFRcreat, and clinical laboratory test values from baseline to Week 12 (safety analysis set)

|  | **Esaxerenone** | | **Trichlormethiazide** | |
| --- | --- | --- | --- | --- |
|  | *n* | Mean ± SD | *n* | Mean ± SD |
| **UA (mg/dL)** |  |  |  |  |
| Baseline | 302 | 5.39 ± 1.27 | 297 | 5.41 ± 1.20 |
| Week 2 | 295 | 5.73 ± 1.32 | 295 | 6.02 ± 1.44 |
| Change from baseline | 295 | 0.36 ± 0.58 | 294 | 0.61 ± 0.86 |
| Week 4 | 295 | 5.73 ± 1.33 | 291 | 6.02 ± 1.35 |
| Change from baseline | 295 | 0.34 ± 0.67 | 290 | 0.62 ± 0.70 |
| Week 8 | 287 | 5.81 ± 1.29 | 288 | 6.06 ± 1.30 |
| Change from baseline | 287 | 0.41 ± 0.66 | 287 | 0.65 ± 0.77 |
| Week 12 | 283 | 5.84 ± 1.34 | 284 | 6.06 ± 1.41 |
| Change from baseline | 283 | 0.43 ± 0.75 | 283 | 0.66 ± 0.96 |
| **Serum Na (mEq/L)** |  |  |  |  |
| Baseline | 302 | 140.99 ± 2.16 | 298 | 141.16 ± 1.97 |
| Week 2 | 295 | 140.27 ± 2.31 | 295 | 140.58 ± 2.27 |
| Change from baseline | 295 | −0.70 ± 2.00 | 295 | −0.57 ± 1.89 |
| Week 4 | 295 | 140.28 ± 2.37 | 291 | 140.68 ± 2.30 |
| Change from baseline | 295 | −0.70 ± 2.02 | 291 | −0.44 ± 1.98 |
| Week 8 | 287 | 140.45 ± 2.32 | 288 | 140.63 ± 2.38 |
| Change from baseline | 287 | −0.54 ± 2.02 | 288 | −0.50 ± 1.98 |
| Week 12 | 283 | 140.54 ± 2.42 | 284 | 140.71 ± 2.55 |
| Change from baseline | 283 | −0.44 ± 2.20 | 284 | −0.44 ± 2.18 |
| **Serum K (mEq/L)** |  |  |  |  |
| Baseline | 293 | 4.21 ± 0.35 | 285 | 4.21 ± 0.33 |
| Week 2 | 285 | 4.36 ± 0.35 | 282 | 4.09 ± 0.38 |
| Change from baseline | 284 | 0.15 ± 0.35 | 281 | −0.12 ± 0.32 |
| Week 4 | 286 | 4.28 ± 0.37 | 279 | 4.10 ± 0.36 |
| Change from baseline | 284 | 0.07 ± 0.37 | 278 | −0.11 ± 0.35 |
| Week 8 | 279 | 4.30 ± 0.37 | 276 | 4.05 ± 0.35 |
| Change from baseline | 278 | 0.09 ± 0.38 | 275 | −0.16 ± 0.34 |
| Week 12 | 275 | 4.24 ± 0.34 | 272 | 3.99 ± 0.35 |
| Change from baseline | 274 | 0.04 ± 0.35 | 272 | −0.22 ± 0.35 |
| **Serum Ca (mEq/L)** |  |  |  |  |
| Baseline | 302 | 9.26 ± 0.36 | 295 | 9.25 ± 0.37 |
| Week 2 | 294 | 9.32 ± 0.38 | 295 | 9.33 ± 0.35 |
| Change from baseline | 294 | 0.07 ± 0.32 | 292 | 0.08 ± 0.33 |
| Week 4 | 295 | 9.31 ± 0.38 | 291 | 9.33 ± 0.37 |
| Change from baseline | 295 | 0.06 ± 0.30 | 288 | 0.07 ± 0.34 |
| Week 8 | 287 | 9.28 ± 0.39 | 288 | 9.30 ± 0.38 |
| Change from baseline | 287 | 0.03 ± 0.32 | 285 | 0.04 ± 0.34 |
| Week 12 | 283 | 9.26 ± 0.37 | 284 | 9.30 ± 0.37 |
| Change from baseline | 283 | 0.01 ± 0.32 | 281 | 0.05 ± 0.37 |
| **Serum Cl (mEq/L)** |  |  |  |  |
| Baseline | 302 | 103.67 ± 2.60 | 298 | 103.75 ± 2.48 |
| Week 2 | 295 | 103.22 ± 2.68 | 295 | 102.32 ± 2.69 |
| Change from baseline | 295 | −0.45 ± 2.03 | 295 | −1.46 ± 2.26 |
| Week 4 | 295 | 103.20 ± 2.66 | 291 | 102.46 ± 2.50 |
| Change from baseline | 295 | −0.47 ± 2.22 | 291 | −1.31 ± 2.17 |
| Week 8 | 287 | 103.73 ± 2.65 | 288 | 102.44 ± 2.60 |
| Change from baseline | 287 | 0.02 ± 2.23 | 288 | −1.33 ± 2.37 |
| Week 12 | 283 | 104.00 ± 2.63 | 284 | 102.60 ± 2.77 |
| Change from baseline | 283 | 0.31 ± 2.22 | 284 | −1.17 ± 2.65 |
| **eGFRcreat (mL/min/1.73m^2^)** |  |  |  |  |
| Baseline | 302 | 71.48 ± 15.75 | 295 | 71.95 ± 17.12 |
| Week 2 | 295 | 66.28 ± 14.76 | 295 | 68.79 ± 15.91 |
| Change from baseline | 295 | −5.21 ± 8.07 | 292 | −3.24 ± 8.84 |
| Week 4 | 295 | 66.41 ± 15.08 | 291 | 68.28 ± 15.76 |
| Change from baseline | 295 | −5.09 ± 8.23 | 288 | −3.79 ± 8.80 |
| Week 8 | 287 | 65.30 ± 15.52 | 288 | 67.72 ± 16.10 |
| Change from baseline | 287 | −6.17 ± 8.78 | 285 | −4.48 ± 9.65 |
| Week 12 | 283 | 64.44 ± 14.77 | 284 | 67.61 ± 15.37 |
| Change from baseline | 283 | −7.08 ± 8.61 | 281 | −4.67 ± 9.84 |
| **HbA1c (%)** |  |  |  |  |
| Baseline | 301 | 6.23 ± 0.99 | 296 | 6.19 ± 0.94 |
| Week 12 | 282 | 6.29 ± 1.09 | 283 | 6.27 ± 0.98 |
| Change from baseline | 281 | 0.08 ± 0.47 | 281 | 0.07 ± 0.42 |
| **Serum glucose (mg/dL)** |  |  |  |  |
| Baseline | 302 | 121.3 ± 49.6 | 297 | 117.1 ± 43.3 |
| Week 12 | 281 | 121.0 ± 44.4 | 283 | 122.8 ± 45.7 |
| Change from baseline | 281 | 0.1 ± 39.6 | 282 | 5.8 ± 45.0 |

*P*-values were not calculated for changes from baseline to Week 12.

*Ca* calcium, *Cl* chloride, *eGFRcreat* creatinine-based estimated glomerular filtration rate*, HbA1c* glycated hemoglobin, *K* potassium, *Na* sodium, *SD* standard deviation, *UA* uric acid.

**Supplementary Table 9.** Incidence of serum K level <3.5, ≥5.5, and ≥6.0 mEq/L (safety analysis set)

| **Serum K level** | **Esaxerenone**  ***n* = 295** | **Trichlormethiazide**  ***n* = 287** |
| --- | --- | --- |
| Serum K <3.5 mEq/L | 9 (3.1) [1.4, 5.7] | 33 (11.5) [8.0, 15.8] |
| Serum K ≥5.5 mEq/L | 6 (2.0) [0.7, 4.4] | 2 (0.7) [0.1, 2.5] |
| Serum K ≥6.0 mEq/L | 0 (0.0) [0.0, 1.2] | 0 (0.0) [0.0, 1.3] |

Data are *n* (%) [95% CI].

*CI* confidence interval, *K* potassium.

**Supplemental Figures**


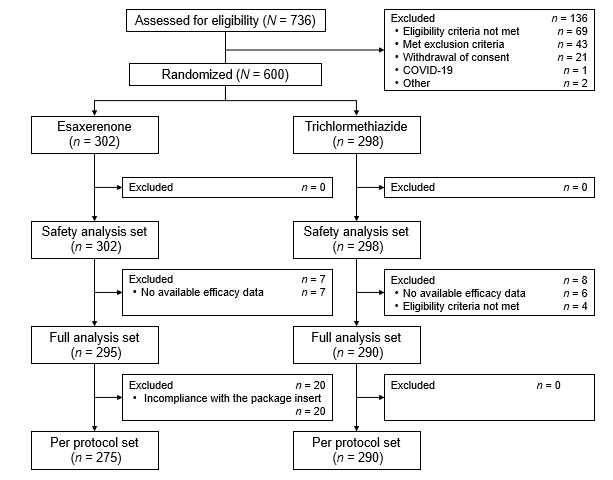


**Supplementary Fig. 1.** Patient disposition
